# Phase Lag Index of Resting-State EEG for Identification of Mild Cognitive Impairment Patients with Type 2 Diabetes

**DOI:** 10.3390/brainsci12101399

**Published:** 2022-10-17

**Authors:** Yuxing Kuang, Ziyi Wu, Rui Xia, Xingjie Li, Jun Liu, Yalan Dai, Dan Wang, Shangjie Chen

**Affiliations:** 1 The Second School of Clinical Medicine, Southern Medical University, Guangzhou 510515, China; 2 Department of Rehabilitation, Affiliated Baoan Hospital of Shenzhen, Southern Medical University (The People’s Hospital of Baoan Shenzhen), Shenzhen 518101, China

**Keywords:** mild cognitive impairment, diabetes, resting-state EEG, phase lag index

## Abstract

Mild cognitive impairment (MCI) is one of the important comorbidities of type 2 diabetes mellitus (T2DM). It is critical to find appropriate methods for early diagnosis and objective assessment of mild cognitive impairment patients with type 2 diabetes (T2DM-MCI). Our study aimed to investigate potential early alterations in phase lag index (PLI) and determine whether it can distinguish between T2DM-MCI and normal controls with T2DM (T2DM-NC). EEG was recorded in 30 T2DM-MCI patients and 30 T2DM-NC patients. The phase lag index was computed and used in a logistic regression model to discriminate between groups. The correlation between the phase lag index and Montreal Cognitive Assessment (MoCA) score was assessed. The α-band phase lag index was significantly decreased in the T2DM-MCI group compared with the T2DM-NC group and showed a moderate degree of classification accuracy. The MoCA score was positively correlated with the α-band phase lag index (r = 0.4812, moderate association, *p* = 0.015). This work shows that the functional connectivity analysis of EEG may offer an effective way to track the cortical dysfunction linked to the cognitive deterioration of T2DM patients, and the α-band phase lag index may have a role in guiding the diagnosis of T2DM-MCI.

## 1. Introduction

Type 2 diabetes mellitus (T2DM) is a long-term metabolic illness defined by improper glucose metabolism and insulin resistance. It has a wide range of physiological side effects, including those that affect the central nervous system [1]. The increased tau hyperphosphorylation and intra-neuronal β-amyloid deposition in the brain are results of insulin resistance and hyperinsulinemia [2]. These insulin-related effects may have an impact on cognitive function and may hasten the onset of mild cognitive impairment (MCI) [3]. MCI is seen as a transitional stage between healthy aging and dementia [4]. MCI patients exhibit memory impairment, but their functional capacity remains. T2DM is linked to an increase in the percentage of individuals who transition from MCI to dementia [5], while previous research showed that the conversion rate is 1.5–3 times higher than people without T2DM [6]. More importantly, cognitive dysfunction such as memory and learning ability decline will cause the decline of self-management ability and deterioration of glycemic control in T2DM patients, which not only affects their quality of life but also brings a heavy economic burden to society. Since MCI is one of the important comorbidities of T2DM [7], it is critical to find appropriate methods for the early diagnosis and objective assessment of mild cognitive impairment patients with type 2 diabetes (T2DM-MCI).

Electroencephalography (EEG) is an inexpensive and non-invasive method with high temporal resolution [8]. Recent studies have shown new ways of EEG applications, such as sleep stages [9], driving workload [10], and brain stimulation for different neurological workloads [11]. Although EEG has been used for many years, using it as a cognitive biomarker to identify and forecast diseases is a relatively new endeavor. Several studies have used machine learning methods for stroke prediction, describing the classification of stroke-derived cognitive impairment using EEG [12,13]. There is growing evidence that EEG biomarkers can be used to identify early abnormalities in neuronal function before cortical tissue loss or cognitive decline. Many researchers believe that the disruption of functional connectivity may be a pathological characteristic of neurodegenerative diseases [14]. Several studies used coherence as a method of functional connectivity and found a significant reduction in α-band coherence in AD patients [15,16]. According to Musaeus et al. (2019), there was a considerable decline in the α-band EEG synchronization in MCI patients, and this decline was correlated with the degree of cognitive impairment as determined by the Mini-Mental State Examination (MMSE) [17]. The results mentioned above imply that reduced α-band functional connectivity represents particular pathophysiology in MCI and AD patients.

Notably, phase synchronization is a way of functional connectivity representation that may show functional connectivity across various cortical areas of the brain and reveal information about the synchronization of the regional cortical activity [18]. The phase lag index (PLI) is an important index of phase synchronization. The measurement of the phase lag index can be used to quantify changes in functional connectivity indirectly as a result of disease progression [19,20]. The main advantage of the phase lag index over other functional connectivity measures is that it is insensitive to the effects of volume conduction [21].

Functional connectivity measures of EEG may serve as features for a diagnostic classifier. In this study, we investigated the potential early alteration of the phase lag index and tested the classification feature of the phase lag index to determine whether it could distinguish between T2DM-MCI and normal controls with T2DM (T2DM-NC) patients. We also identified the correlation between the phase lag index and neuropsychological tests to figure out whether phase lag index characteristics could reflect cognitive degeneration in T2DM-MCI patients. We hypothesized that the phase lag index is decreased in T2DM-MCI patients compared to T2DM-NC patients and that this indicator is associated with cognitive function in T2DM-MCI patients.

This study looked for biomarkers for early diagnosis of T2DM-MCI patients and revealed the role of phase lag index in differentiating T2DM-MCI and T2DM-NC patients.

This study explored the diagnostic accuracy of the phase lag index between T2DM-MCI and T2DM-NC patients.

This study explored the association between phase lag index and cognitive function.

## 2. Materials and Methods

### 2.1. Participants

Our study enrolled 30 T2DM-MCI patients and 30 T2DM-NC patients who were matched for age, sex, and educational level from the Affiliated Baoan Hospital of Shenzhen, Southern Medical University. Cognitive deterioration was assessed by an experienced physician who works in cognitive rehabilitation. The inclusion criteria of the T2DM-MCI group include: (1) satisfied the diagnostic criteria for T2DM (The Chinese Guidelines for the Prevention and Treatment of Type 2 Diabetes, 2019) [22]; (2) met the MCI diagnostic criteria (Petersen, 2004) [23]; (3) over 60 years old. The inclusion criteria of the T2DM-NC group include: (1) satisfied the diagnostic criteria for T2DM (The Chinese Guidelines for the Prevention and Treatment of Type 2 Diabetes, 2019) [22]; (2) did not meet the MCI diagnostic criteria (Petersen, 2004) [23]; (3) over 60 years old. The exclusion criteria include: (1) neuropsychosis and other medical or neuropsychological conditions such as alcohol and/or substance abuse that can cause brain dysfunction; (2) combined with severe primary disorders such as those of the cardiovascular, cerebrovascular, liver, kidney, and hematological system; (3) take cognition-related drugs or participate in other similar research projects; (4) epilepsy or epilepsy family, hypothyroidism, mental illness in the past or several other factors that might influence cognitive function; (5) subjects with severe decreases in vitamin B12 as a result of pharmacological treatment with metformin; (6) subjects have a parental history of dementia. The Affiliated Baoan Hospital of Shenzhen, Southern Medical University’s Ethics Committee for Clinical Research approved this study. All participants provided written informed consent after being informed about the study.

### 2.2. EEG Recording and Processing

EEG data were collected from 64 scalp electrodes placed according to the international extended 10–20 system using the Neuroscan system (Neuvo 64, Neuroscan Compumedics, Australia) at the Department of Rehabilitation of the Affiliated Baoan Hospital of Shenzhen, Southern Medical University. Subjects were asked to be eyes closed, awake, and quiet for five minutes during EEG acquisition. In order to search for eye-blinking artifacts, vertical and horizontal electrooculography (EOG) channels were located. The impedance of all electrodes was maintained below 5 KΩ, and the sampling rate was 1000 Hz.

The preprocessing process was as follows. Firstly, eye electrodes HEO and VEO were removed, and re-referencing was completed using the average of the left and right mastoid sensors. Next, the recorded EEG data were bandpass filtered to 0.1–30 Hz, and a notch filter was used to remove the line noise between 48 and 52 Hz. Afterward, the sampling rate was down-sampled to 500 Hz and EEG data were segmented into 3 s epochs. Then, independent component analysis was used to reject other artifact components, including EOG, electromyography (EMG), and electrocardiogram (ECG) artifacts. Then, the remaining EEG data were checked manually, and data segments with muscular and cardiac artifacts were removed.

The phase lag index was used to calculate the functional connectivity between several brain areas [24]. The phase lag index measures the asymmetry in the distribution of phase discrepancies between two signals. It displays the consistency of the phase lead or lag of one signal to another. For example, if the phase differences between two-time series are △*ϕ*(*t*_k_)(k = 1 …N), then the phase lag index can be calculated by:

PLI = |〈sign[△*ϕ*(*t*_k_)]〉|

where sign stands for signum, sin stands for sinusoidal function, and < > and | | stand for mean and absolute value, respectively. The phase lag index has a value between 0 and 1, with 0 denoting complete synchronization and 1 denoting perfect non-zero phase locking [25]. Node-to-node phase lag index values were computed in the four frequency bands: delta (1–4 Hz), theta (4–8 Hz), alpha (8–13 Hz), and beta (13–30 Hz). A 62 × 62 connectivity matrix was created by treating each EEG channel signal as a real-valued time series. The average phase lag index across all node pairs was also calculated and utilized as a global EEG synchronization measure.

### 2.3. Statistical Analysis

The data were analyzed using SPSS 27.0 (IBM Corp. Released 2020. IBM SPSS Statistics for Windows, Version 27.0. Armonk, NY, USA: IBM Corp). All data were tested for normality. When the data conformed to a normal distribution, they were expressed as mean and standard deviation (SD). An independent sample *t*-test and *χ^2^* test were used to compare the clinical characteristics between T2DM-MCI and T2DM-NC groups. Data that were not normally distributed were analyzed using the Wilcoxon rank sum test. The diagnostic model was created using multi-factor binary logistic regression analysis, and the diagnostic model’s discriminatory power was assessed using the area under the curve (AUC). The diagnostic model was considered to have good discriminatory ability when the AUC value was > 0.75. The AUC of the phase lag index was counted, and the receiver operating characteristic (ROC) curves were plotted. The *p*-value was set at 0.05 for the significance level.

For the purpose of classifying T2DM-MCI and T2DM-NC patients, the accuracy of the phase lag index was estimated using machine learning analysis of logistic regression. The samples were divided into test and training sets by 10-fold cross-validation (1 fold for testing and the remaining 9 folds for training). The training set data were used to train on the logistic regression classifier, and the test set data were used to make predictions and to calculate the accuracy, specificity, sensitivity, precision, F1 value, and AUC to assess the overall performance of the logistic regression model.

## 3. Results

### 3.1. Subject Characteristics

The clinical characteristics such as the participants’ gender, age, education level, and overall cognitive states are shown in the table below (See Table 1). The results showed that the two groups were matched in gender, age, and education level. Additionally, there was a significant difference between the Montreal Cognitive Assessment (MoCA) score of the T2DM-MCI group and the T2DM-NC group (*p* < 0.001).

### 3.2. Electroencephalographic Results

**Figure 1:**
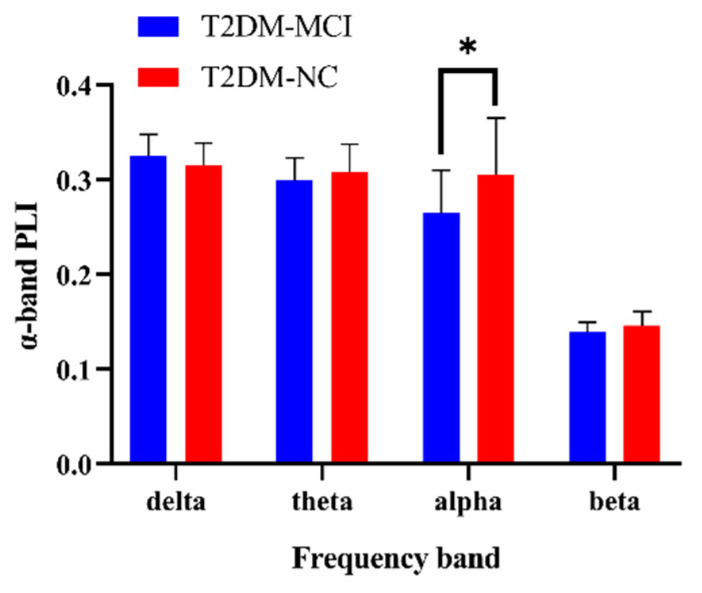
The phase lag index of all pairs of EEG channels in four frequency bands between T2DM-MCI and T2DM-NC patients. The T2DM-MCI group and T2DM-NC group are shown in blue and green boxes, respectively. Error bars are standard deviations. * *p* < 0.05.

In each of the four frequency bands, the phase lag index of every pair-wise combination of channels was calculated independently. The phase lag index in each frequency band was compared between T2DM-MCI and T2DM-NC groups using the Wilcoxon rank sum test. The findings of between-group variations in the phase lag index are displayed in Figure 1. Compared with the T2DM-NC group, the α-band phase lag index in the T2DM-MCI group was significantly decreased. The outcomes of the additional frequency bands did not differ appreciably. Compared with T2DM-NC patients, the phase lag index in T2DM-MCI patients was marginally lower in the beta and theta bands and marginally higher in the delta band. The multi-factor binary logistic regression analysis results are presented in the Supplementary Material.

**Figure 2:**
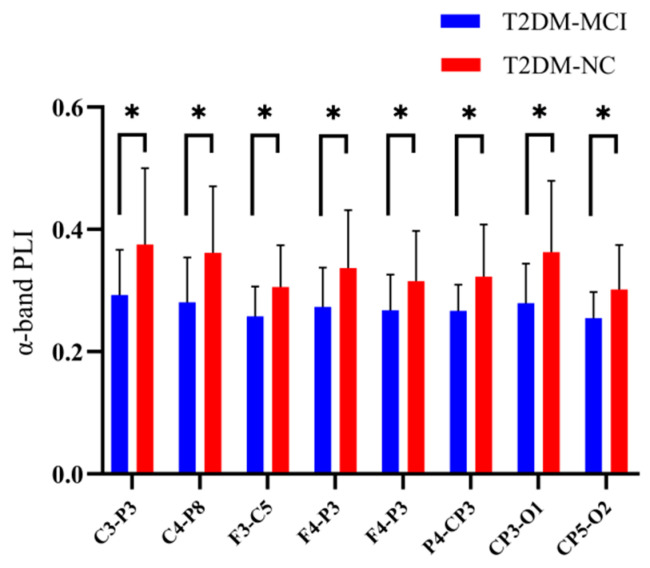
The phase lag index of eight specific electrode pairs in the alpha band between the T2DM-MCI and T2DM-NC groups. The T2DM-MCI group and T2DM-NC group are shown in blue and green boxes, respectively. Error bars are standard deviations. * *p* < 0.05.

The difference in the α-band phase lag index between different electrode pairs in the T2DM-MCI group and the T2DM-NC group was further analyzed, and the results are shown in Figure 2. The eight specific electrode pairs included: F4-P3, P4-CP3, and CP5-O2 in inter-hemispheric regions, and C3-P3, C4-P8, F3-C5, F8-P8, and CP3-O1 in intra-hemispheric regions. The results showed that the α-band phase lag index of T2DM-MCI patients was significantly lower than that of T2DM-NC patients in both inter-hemispheric and intra-hemispheric regions.

**Figure 3:**
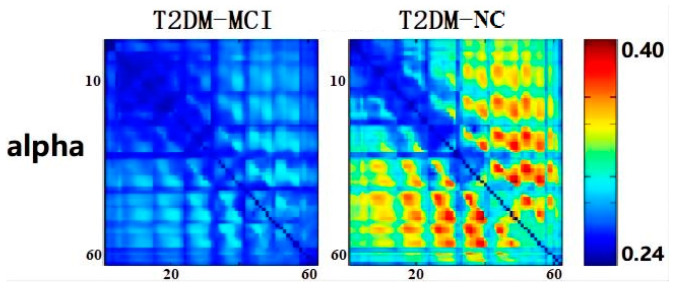
The alpha band matrix of phase lag index in the T2DM-MCI group (**left**) and the T2DM-NC group (**right**). Here, 1 to 62 electrodes are shown from left to right, top to bottom. The color bars indicates the phase lag index values.

Figure 3 shows the alpha band matrix of phase lag index in the T2DM-MCI group and the T2DM-NC group. The matrices of the two groups were clearly different, as can be observed. The T2DM-NC group (right) showed more areas of high values (red) than the T2DM-MCI group (left). In the T2DM-NC group, the high values of phase lag index were distributed in parietal and parieto-occipital areas (C3/C4-P3/P4, P3/P4-O1/O2) in the alpha band.

### 3.3. Machine Learning Applications

**Figure 4:**
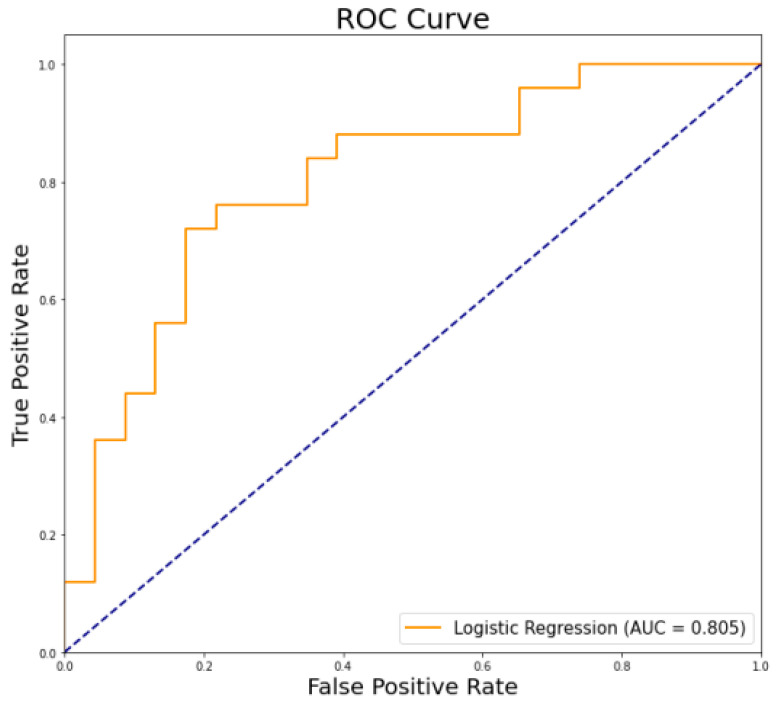
ROC curve was obtained by the logistic regression classifier.

The α-band phase lag index measures were used to train the logistic regression classifier. The ROC curve was obtained by the logistic regression classifier. As can be seen from Figure 4, the ROC curve of the α-band phase lag index has an area under the curve of 0.805. In Table 2, the accuracy rate is 75.00%, which is higher than the empirical chance level accuracy of 64.58%, proving that the logistic regression model has a good classification effect.

### 3.4. Correlation Analysis

**Figure 5:**
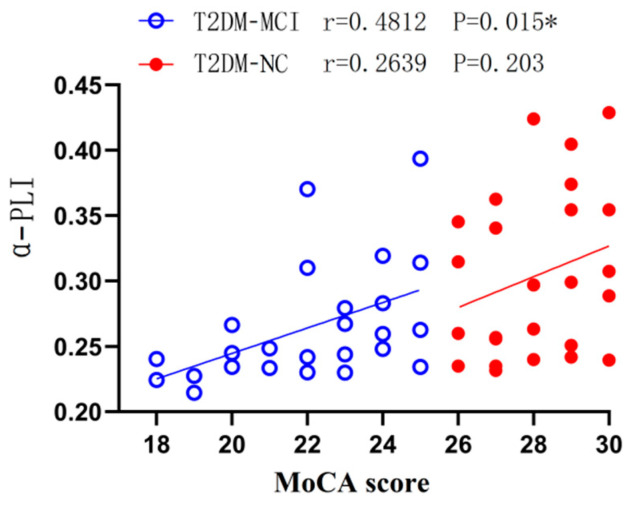
The linear correlation between the phase lag index and the MoCA score in the alpha band. The findings revealed a significant positive correlation between the MoCA score and the α-band phase lag index among T2DM-MCI patients (r = 0.4812, moderate association, *p* = 0.015). There was no significant correlation in T2DM-NC patients (r = 0.2639, weak association, *p* = 0.203). * *p* < 0.05.

Pearson’s linear correlation was calculated to test whether there was a correlation between the α-band phase lag index and cognitive state. Figure 5 illustrated the correlation between the MoCA score distribution and the α-band phase lag index through a scatter plot. The results showed that the MoCA score was positively correlated with the α-band phase lag index in the T2DM-MCI group (r = 0.4812, moderate association, *p* = 0.015). However, the positive correlation was not significant in the T2DM-NC group (r = 0.2639, weak association, *p* = 0.203). The results suggested that the α-band phase lag index may reflect cognitive dysfunction in T2DM-MCI patients.

## 4. Discussion

### 4.1. This Work

This study aimed to investigate potential early alterations in phase lag index and determine whether it can distinguish between T2DM-MCI and T2DM-NC patients. We discovered that the α-band phase lag index was significantly reduced in the T2DM-MCI group compared with the T2DM-NC group, especially in parietal and parieto-occipital areas. This result suggested that T2DM-MCI patients had impaired functional connectivity, which lent credence to the idea that T2DM-MCI is a disconnection syndrome. Machine learning results showed a moderate degree of classification accuracy (AUC > 0.7) between the two groups. Furthermore, our study also revealed a substantial positive correlation between the MoCA score and the α-band phase lag index. The results suggested that the α-band phase lag index characteristics could reflect the degeneration of cognitive function in T2DM-MCI patients. A comparative analysis of the proposed work and previous works is presented in Table 3.

It is suggested that T2DM is associated with an increased risk of dementia [5], and it is yet unclear how T2DM affects cognition in specific ways. Recent studies revealed that glycemic variability and prediabetes may be a factor associated with hippocampal hypoperfusion and cause cognitive deterioration [26]. Our results showed that the α-band phase lag index was significantly reduced in the T2DM-MCI group compared with the T2DM-NC group, which is in agreement with another phase lag index study [27]. Similarly, in a coherence study, the α-band coherence in the frontal–occipital and temporal–occipital regions was shown to be lower in the T2DM-MCI patients than in the T2DM-NC patients [28]. The results mentioned above implied that reduced alpha functional connectivity may reflect specific pathology in T2DM-MCI patients.

Furthermore, to evaluate the effectiveness of the EEG α-band phase lag index in distinguishing between T2DM-MCI and T2DM-NC patients, we employed a machine learning classification method with a logistic regression classifier. It has been shown that when the AUC is greater than 0.7, the significance of the classification is more pronounced and the diagnosis is better [29]. In the current investigation, we report a moderate accuracy of 75.00% for the individual classification of T2DM-MCI versus T2DM-NC. According to these findings, there is a moderate degree of discrimination between T2DM-MCI and T2DM-NC individuals using the α-band phase lag index.

Our study revealed a moderate association between the MoCA score and the α-band phase lag index in T2DM-MCI patients. This result confirmed the viability and importance of our investigation that intended to identify EEG biomarkers for T2DM-MCI. According to the above findings, the functional connectivity decline between brain channels or regions is linked to cognitive impairment. This association may be the result of a reduction in the processing of local information brought on by synaptic degeneration and the death of cortical neurons, which leads to a gradual loss of connectivity between some cortical areas [30].

**Table 3:** Comparative study of methodologies and results between proposed work and previous works.

Study	Study Sample	EEG Features of Neurological Outcome	Main Findings
Zeng et al. [27]	Sixteen T2DM-aMCI patients and twelve T2DM-NC patients	Phase lag index, clustering coefficient, and path length	The complex network-derived biomarkers based on EEG could be employed to track the cognitive function of diabetic patients and provide a new diagnostic tool for T2DM-aMCI patients.
Bian et al. [28]	Sixteen T2DM-aMCI patients and twelve T2DM-NC patients	Relative power and coherence	The decreased theta, alpha coherence, and increased delta coherence in corresponding regions may distinguish T2DM-aMCI from T2DM-NC and help the diagnosis of T2DM-aMCI patients.
Wen et al. [31]	Nineteen T2DM-aMCI patients and twenty T2DM-NC patients	Permutation conditional mutual information (PCMI)	The coupling strength or directionality of EEG signals calculated by PCMI might be used as a biomarker in distinguishing the T2DM-aMCI from T2DM-NC.
Cui et al. [32]	Eight T2DM-aMCI patients and eleven T2DM-NC patients	Synchronization index (SI) and global synchronization index (GSI)	Each of the methods reflected that the cortical source synchronization was significantly different between the aMCI and the control group, and these differences correlated with cognitive functions.
Lu et al. [33]	Seventeen T2DM-aMCI patients and ten T2DM-NC patients	Correlation between probabilities of recurrence (CPR)	The synchronization value of the EEG signal was significantly decreased in T2DM-aMCI patients compared with T2DM-NC patients, and the EEG indicator was associated with cognitive impairment in T2DM-aMCI patients.
Proposed work	Thirty T2DM-MCI patients and thirty T2DM-NC patients	Phase lag index	The functional connectivity analysis of EEG may offer an effective way to track the cortical dysfunction linked to the cognitive deterioration of T2DM patients, and the α-band phase lag index may have a role in guiding the diagnosis of T2DM-MCI.

### 4.2. Contributions

In this work, the potential biomarker for differentiating T2DM-MCI and T2DM-NC groups was explored using the α-band phase lag index. The results of the logistic regression classifier may shed light on the use of machine learning techniques in the diagnosis of T2DM-MCI.

### 4.3. Limitations

This study has several limitations. Firstly, this was a cross-sectional study. Future work could consider collecting longitudinal data to monitor the predictive value of resting-state EEG characteristics in T2DM-MCI participants. Secondly, no restriction was imposed on the range of education levels, and the severity of cognitive impairment in T2DM-MCI patients was not controlled. Further studies with large populations are needed to distinguish T2DM patients with different levels of education and different levels of cognitive impairment. Thirdly, given the relatively long time required for neuropsychological testing, machine learning methods using resting-state EEG functional connectivity features may be thought of as a practical screening tool to distinguish T2DM-MCI from T2DM-NC. However, the α-band phase lag index explains only a small fraction of the neural correlates of overall cognitive function in T2DM-MCI and T2DM-NC patients. Therefore, future studies could use other functional connectivity metrics such as coherence, synchronization possibilities, and global field synchronization to complement the resting-state EEG results.

### 4.4. Future Work

Firstly, except for monitoring EEG during the resting state, we can also record EEG during cognitive tasks, including attentional, episodic, and working memory tasks. Secondly, future prospective research involving sizable populations is required to ascertain whether the machine learning algorithms could distinguish between T2DM-MCI and T2DM-NC, which may offer new information about the efficacy of machine learning applications for categorizing cognitive decline in T2DM patients.

## 5. Conclusions

This work shows that the functional connectivity analysis of EEG may offer an effective way to track the cortical dysfunction linked to the cognitive deterioration of T2DM patients. The α-band phase lag index shows a moderate degree of classification accuracy (AUC > 0.7) between T2DM-MCI and T2DM-NC patients. These findings suggested that the α-band phase lag index may be used as a supplementary diagnostic tool and may eventually play a role in guiding the diagnosis of T2DM-MCI. Based on these preliminary findings, in future studies, we should confirm the effectiveness of machine learning approaches to identify T2DM-MCI patients in prospective trials with large samples.

## Figures and Tables

**Table 1:** Clinical characteristics of the participants.

Group	T2DM-MCI (*N* = 30)	T2DM-NC (*N* = 30)	*χ*^2^/*t* Value	*p*-Value
Gender (M/F)	13/17	16/14	0.601	0.438
Age (years)	67.17 ± 4.12	67.73 ± 4.40	−0.515	0.609
Education level (years)	10.63 ± 3.75	10.23 ± 3.23	0.443	0.660
MoCA(scores)	22.08 ± 2.24	28.08 ± 1.41	−11.350	<0.001 *
Duration of T2DM (years)	14.23 ± 7.80	15.43 ± 8.34	−0.576	0.567
Duration of MCI (years)	3.33 ± 1.86	-	-	-

The data are displayed as mean ± SD. The *p*-value for gender was discovered using the chi square test, while *p*-values for the comparison of demographic information and neuropsychological performance were discovered using an independent sample t-test. M, male; F, female; SD, standard deviation; MoCA, Montreal Cognitive Assessment; T2DM, type 2 diabetes mellitus; MCI, mild cognitive impairment. * *p* < 0.05.

**Table 2:** Accuracy, empirical chance level, specificity, sensitivity, precision, F1 value, and AUC using logistic regression classifier.

Classifier	Accuracy (%)	Empirical Chance Level Accuracy (%)	Specificity (%)	Sensitivity (%)	AUC
Logistic Regression	75.00	64.58	60.87	88.00	0.805

Empirical chance level accuracy is the 95th percentile of the empirical performance distribution created by randomly permuting the labels 5000 times, representing the original classification as significant at *p* < 0.05.

## Data Availability

The data that support the findings of this study are available from the corresponding author upon reasonable request.

## Supplementary Materials

The following supporting information can be downloaded at: https://www.mdpi.com/article/10.3390/brainsci12101399/s1, Figure S1: ROC curves from different frequency bands. Performance is good when the curves are around the upper left corner (0.0, 1.0).

## Abbreviations

Abbreviations	Full Name
T2DM	Type 2 Diabetes Mellitus
MCI	Mild Cognitive Impairment
T2DM-MCI	Mild Cognitive Impairment patients with Type 2 Diabetes
EEG	Electroencephalography
AD	Alzheimer’s Disease
MMSE	Mini-Mental State Examination
PLI	Phase Lag Index
T2DM-NC	Normal Controls with Type 2 Diabetes
EOG	Electrooculography
EMG	Electromyography
ECG	Electrocardiogram
SD	Standard Deviation
AUC	Area Under Curve
ROC	Receiver Operating Characteristic
MoCA	Montreal Cognitive Assessment
SVM	Support Vector Machine
LDA	Linear Discriminant Analysis
PCMI	Permutation Conditional Mutual Information
SI	Synchronization Index
GSI	Global Synchronization Index
CPR	Correlation between Probabilities of Recurrence
