# Supplementary material for: Phase Lag Index of Resting-State EEG for Identification of Mild Cognitive Impairment Patients with Type 2 Diabetes
Source: Brain Sci. 2022 Oct 17;12(10):1399. doi: 10.3390/brainsci12101399 (PMC9599801; doi:10.3390/brainsci12101399)
Supplement: Supplementary file 1 [file brainsci-12-01399-s001.zip › brainsci-1937059-supplementary.pdf]

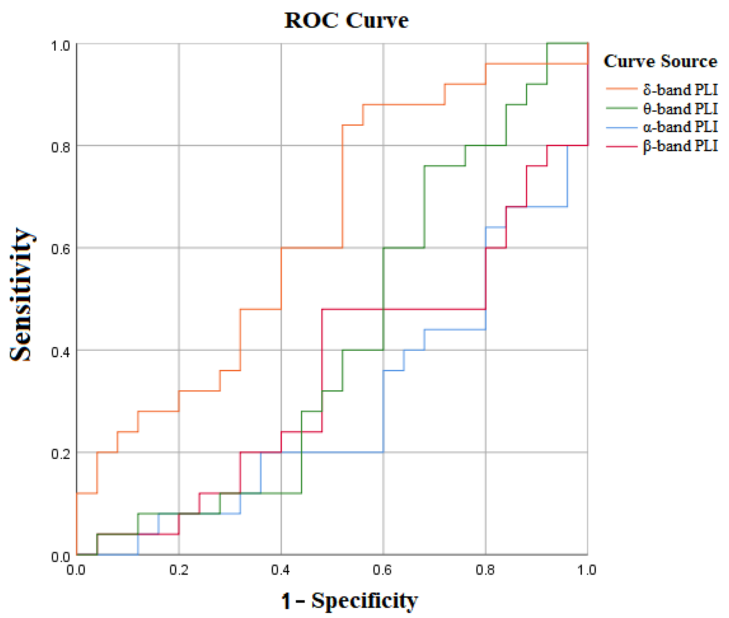

Figure S1. ROC curves from different frequency bands. Performance is good when the curves are around the upper left corner (0.0, 1.0).

The model's ROC curves can be used to compare the degree of disease recognition performance of two or more various diagnostic approaches. The advantages and drawbacks of each diagnostic approach can be seen graphically by plotting the ROC curves of each diagnostic method in the same ROC space (inside the same coordinate system). The diagnostic approach performs better as indicated by the ROC curve that is closer to the upper left corner. Here, we analyze four eigenvalues for the ROC curve. As shown in the figure above, it can be seen that δ-band PLI has the best diagnostic performance, followed by θ-band PLI.

		Predicted Classes		
		T2DM-MCI	T2DM-NC	
True Classes	T2DM-MCI	22	3	88.0%
	T2DM-NC	1	24	96.0%
				Overall Accuracy: 92.0%

The above table shows an overall accuracy rate of 92%.
